# Intrauterine Fetal Gene Therapy: Is That the Future and Is That Future Now?

**DOI:** 10.7759/cureus.22521

**Published:** 2022-02-23

**Authors:** Nikhil Chowdary Peddi, Harshita Marasandra Ramesh, Sai Sravya Gude, Sai Sreeya Gude, Sravya Vuppalapati

**Affiliations:** 1 Pediatrics, PES Institute of Medical Sciences and Research, Kuppam, IND; 2 Pediatrics, Kasturba Medical College, Mangalore, Mangalore, IND; 3 Pediatrics, Guntur Medical College, Guntur, IND

**Keywords:** prenatal gene therapy, crispr/cas9 gene editing, prenatal genetic testing, fetal therapeutics, viral and non-viral vectors

## Abstract

Researchers are looking into techniques to intervene sooner and earlier in the disease process thanks to advances in disease genetics, etiologies, and prenatal diagnosis. We conducted a literature search in PubMed-indexed journals to provide an overview of the evolution of gene therapy, rationale for prenatal gene therapy, uses and risks of gene therapy, and ethical issues following the usage of gene therapy. Recent animal research has revealed that transmitting genetic material to a growing fetus through viral and non-viral vectors is conceivable besides proving how gene-editing technology is achieved by various mechanisms that utilize zinc finger nucleases, TAL effector nucleases, and clustered short palindromic repeats-Cas9 complex. This review offers an overview of the current knowledge in the field of prenatal gene therapy, as well as potential future research avenues. In addition, it weighs the risks of prenatal gene therapy, such as oncogenesis, genetic mutation transfer from mother to child, and fetal disruption, against the expected benefits, such as preventing the development of severe early-onset illness symptoms, targeting previously inaccessible organs, and establishing tolerance to the therapeutic transgenic protein, all of which lead to permanent somatic gene correction.

This review discusses the scientific, ethical, legal, and sociological implications of these groundbreaking genetic disease prevention techniques, as well as the parameters that must be satisfied for a future clinical application to be considered.

## Introduction and background

Gene therapy involves delivering genetic material to a cell to elicit a therapeutic effect, such as correcting an error or giving cells a new function. This is accomplished by using a vector to deliver the genes to the correct cell, where the expression of the protein produces a therapeutic effect [1,2].

Gene therapy became popular in the 1980s, and the first human gene therapy trials began more than a decade ago. However, despite ongoing technological advancements, most clinical outcomes have been poor. There are several reasons for this, including difficulty in identifying the proper organ, a strong immunological reaction to therapy in adults, and low levels of expression of therapeutic gene products. Many of these issues can be avoided if therapy is applied to the fetus [1,3].

Prenatal gene therapy has been proposed for life-threatening diseases for which prenatal gene delivery offers an advantage over cell transplantation or postnatal gene therapy, and for which satisfactory treatments are currently not available [3]. A growing body of experimental data shows that fetal gene therapy has a therapeutic potential [1,4,5]. This review presents our perspective on the evolution, uses, risks, current state, and future applications of gene therapy in the treatment of congenital genetic disorders.

## Review

Evolution of gene therapy

Role of Vectors in Fetal Gene Therapy

The transfer of genes into fetal cells is accomplished with the help of vectors [6,7]. The choice of vectors depends on the target tissue, the circumstances, and the goals of prenatal treatment [6]. The chosen vector must be safe for both the fetus and the mother, and it should not induce an immune response and oncogenesis. According to the National Institutes of Health (NIH) Recombinant DNA Advisory Committee, the disease of interest should be associated with significant morbidity and mortality for the fetus and should not have available effective postnatal treatments [7].

Types of Vectors

Various types of vectors are used in gene transfer in clinical trials. They can be broadly classified into viral and non-viral vectors [6,8-10]. The choice of vector is influenced by its tissue tropism, packaging ability, immunogenicity, and the ability to produce the vector [6].

Retroviruses: These are ssRNA viruses that integrate with the host genome and have low immunogenicity. Retroviruses were considered successful vectors in animal experiments until the early 2000s. However, research with first-generation gene therapy in children with severe combined immunodeficiency (SCID) revealed that retroviruses are carcinogenic, putting an end to their use as vectors [8].

Lentiviruses: These are a subclass of retroviruses. They are capable of transducing in dividing and quiescent cells [8]. After injecting a lentiviral vector expressing the *beta-globin* gene into humanized mouse models with beta-thalassemia, Shangaris et al. demonstrated that normal hemoglobin levels could be achieved postnatally [11]. However, extensive research is needed to confirm the overall efficacy and safety of these vectors for prenatal gene transfer [8].

Adenoviruses: These are non-enveloped dsDNA viruses with an icosahedral capsule [8]. They remain as episomes without integrating with the host genome. However, their use in in utero gene transfer is limited by their high immunogenicity. However, they are highly favored in adult clinical trials due to persistent gene expression and a wide range of tropisms [6,8].

Adeno-associated viruses (AAVs): These are single-stranded, non-enveloped DNA viruses with an icosahedral capsule. AAV is episomal and can be used in both dividing and non-dividing cells [8]. They are less immunogenic than adenoviruses [8,9]. For example, AAV-mediated intrauterine growth restriction (IUGT) was used in the treatment of Gaucher disease in a mouse model. Repeated studies have demonstrated the efficacy and safety of AAV as a vector for IUGT, but further studies are needed to establish their safety profiles [8]. AAV cells can also be used as episomal vectors in quiescent cells, such as muscles and neurons [9]. The disadvantages of AAV vectors are their low packaging capacity and the long latent period needed for the expression of transduced genes [6].

Delivery of Naked DNA Material

Naked DNA particles can be injected directly into the tissue or organ of interest. However, this method of gene delivery is vulnerable to endonuclease degradation. To improve gene delivery, physical methods such as electroporation, gene guns, ultrasound, and hydrodynamics are used [8,12].

Electroporation: This is a method of applying an electrical voltage to transfer genes by improving cell permeability through the destabilization of the cell membrane. Skin, muscle, and neurons are specific targets employed in this method [8,12]. However, gene transfer to solid organs is not feasible. Takeda et al. restored partial hearing and vestibular function by applying electroporation to transduce genes prenatally in mice [13]. However, ethical considerations regarding the delivery of electric shock in animal studies and the release of immunological cytokines were some of the challenges encountered [8].

Gene gun: Ballistic transfection can be used for gene therapy. In this approach, gold nanoparticles (AuNPs) serve as plasmid DNA (pDNA) carriers. Using this kind of gene delivery, it is possible to transport genes into tissues such as muscle, skin, and liver [14]. AuNPs are low in toxicity, have high absorption efficiency, and are resistant to endosomal lysis. In recent studies, AuNPs have been utilized in gene-editing technology in vivo [8].

Ultrasound-mediated transfection: Ultrasound pulses are used to transfer genes into cells by increasing cell permeabilization [12,14]. It also increases gene expression by 300-fold more than the transfer of naked DNA alone [14]. It has a significant future therapeutic application due to its safe and adaptable profile [12,14].

Hydrodynamic delivery: This method of gene delivery consists of applying a hydrodynamic force to transfer DNA into target cells from circulating blood. This technique is influenced by factors such as the strength of the hydrodynamic force, capillary structure, and the structure of the target tissue. Using this method, gene transfer to muscles and the liver can be carried out by injecting pDNA into the femoral artery with adequate force [14].

The transfer of genes cannot be accomplished by injecting naked DNA without the application of hydrodynamic force because it is rapidly degraded by nucleases and cleared by monocytes and immune cells [8,14]. This necessitated the development of chemical methods in which the desired genes were complexed with either a polymer or lipids. This method ensures cell entry, prevents degradation of the transgene, and overall stability in vivo [14].

Polymers: Various polymer-based nanoparticles, such as polyethyleneimine (PEI), poly-L-lysine (PLL), and chitosan, are used in gene transfer. These polymers encapsulate nucleic acids and ensure the targeted delivery of genes to specific sites. The use of PEI and PLL in prenatal gene therapy is limited due to their cytotoxicity [8]. Poly-b-amino esters are safer biodegradable polymer alternatives capable of gene delivery platforms, both in vitro and in vivo. Poly(lactic-co-glycolic acid) opens new doors of exploration for effective gene editing [8].

Liposomes: Lipid nanoparticles (LNPs) are less immunogenic and are one of the safer modes of gene delivery. Fetal plasma is known to have a higher proportion of high-density lipoprotein and, subsequently, apolipoprotein E (ApoE). LNPs can be of great use, particularly in metabolic and hematopoietic disorders, due to their tendency to accumulate in the liver [8]. LNPs can be used in gene editing, as well as in clustered short palindromic repeats (CRISPR) technology. LNPs can be modified to enhance gene delivery. LNPs consist of four main components, namely, ionizable lipids, phospholipids, cholesterol, and polyethylene glycol (PEG)-lipid conjugates. Phospholipids protect against endosomal lysis and form an integral part of the bilayered structure. While cholesterol promotes fusion with the cell membrane, PEG conjugates improve stability and increase the duration of circulation. Although no in utero studies have been conducted using LNPs, they hold a predominant place in prenatal gene therapy [8].

Gene Editing in Fetal Gene Therapy

Gene editing is used in the treatment of monogenic disorders in fetal gene therapy [6,15]. Gene-editing technology is achieved by various mechanisms that utilize zinc finger nucleases, TAL effector nucleases, and CRISPR-Cas9 complex [6,10]. The CRISPR-Cas9 complex utilizes sgRNA complementary to the genetic sequence of interest and creates double-stranded breaks, which are then repaired either by non-homologous end joining or homology-directed repair [8,10,15]. CRISPR has paved the way for more complex methods of gene editing such as base editing. This technology has been used in preclinical studies that have explored prenatal gene therapies for Leber congenital amaurosis type 10 and Hutchinson-Gilford progeria syndrome [8].

Gene editing can be done using different delivery platforms, such as AAV, with greater packaging ability and higher rates of gene transfection, or physical methods, such as LNPs, electroporation, and AuNPs [8,10]. To date, few studies have been conducted to establish the feasibility of CRISPR-Cas9 technology. For example, SpCas9 was administered using AAV to treat protein C deficiency in animal models of congenital lung disease. In afflicted mouse models, survival outcomes improved [10]. Another study by Riccardi et al. demonstrated improvements in phenotypes and correction rates of 6% and 10% in total bone marrow cells and isolated human placental cells, respectively, following in utero treatment [16].

In utero gene editing can be done at various stages of fetal development: mid-to-late gestation somatic cell gene editing [10] and preimplantation embryonic gene editing [10]. Preimplantation embryonic gene editing affects all cells, including germline cells, in contrast to mid-to-late gestation gene editing [10].

Ex Vivo in Utero Gene Editing

In this method, autologous cells are harvested from the fetus and transplanted back to the fetus after gene editing is done in vitro. While it offers the advantage of not exposing the mother to therapy, it confers certain challenges, such as harvesting and maintaining the differentiation of an adequate number of fetal cells. These challenges can be overcome by hematopoietic stem cell transplantation [10]. It also confers the advantages of being used by those who have preexisting or who develop neutralizing antibodies against viral vectors and a prolonged therapeutic window. Studies by El-Akabawy et al. showed a significant increase in factor VIII activity after transplantation of human placental cells transduced ex vivo using a lentivirus vector harboring the factor VIII transgene [17].

Viral Versus Non-Viral Gene Delivery

Viral vectors have a large packaging ability but carry the risks of immunogenicity and tumorigenesis [8]. Non-viral gene delivery platforms are less immunogenic and are important in in utero gene transfer for gene editing. Non-viral vectors are also more versatile and less expensive to produce on a large scale [8,9,14].

Modes and Timing of Prenatal Gene Therapy

The timing and routes of transduction administration play a key role in the distribution and efficiency of transduction [6,9].

Routes of Administration

Intravenous: Intravenous access to the umbilical vein can be obtained through ultrasound guidance. The umbilical vein also acts as a pathway for the systemic route. Umbilical vein injections can be used to target the liver, which is one of the sites of hematopoiesis in the fetus [8].

Intra-amniotic: The distribution of transduction by intra-amniotic injection is influenced by changes in epithelial differentiation [6]. After the formation of the periderm, this route offers limited access to progenitor cells. It also offers increased distribution of transgenes, which is facilitated by fetal breathing and swallowing movements [6,8]. However, a high dose of transfection is needed due to the dilution of genes in the amniotic cavity, which can be addressed by intratracheal injections [8].

Intracardiac: This route is highly specific but associated with an increased risk of the procedure. Other routes of administration include intramuscular, intraperitoneal under ultrasound guidance, and intraparenchymal injections to target the lungs [8,10].

Factors To Be Kept in Mind for Efficient Gene Delivery

The vector must be safe for both the mother and the fetus. It should reach the target organ after administration and should not be rapidly removed from the bloodstream. After the vector reaches its target organ, it undergoes receptor/ligand-mediated uptake, resists endosomal lysis, and reaches the nucleus to express the protein. Moreover, it should not alter the expression of nearby genes or inactivate transgenes [8].

Timing of Prenatal Gene Delivery

Different routes of administration achieve a highly variable degree of distribution, even when injected at the same gestational age. Stem cells can be easily accessed at an earlier gestational age rather than at later stages, thereby increasing the efficiency of transduction at earlier stages of development [6,9]. For example, transduction of murine skin stem cells can be easily done between eight and ten days of gestation through the intra-amniotic route because, later, keratinization makes the procedure impossible to perform. In addition, murine neurons can be transduced before neural tube closure at E9 [9].

Future of Prenatal Gene Therapy

Prenatal gene therapy can treat monogenic disorders even before the pathology of the disease begins, thereby significantly decreasing morbidity and mortality [8,10]. Several clinical trials in animal models have shown that viral vectors are efficient vehicles for in utero gene transfer. Non-viral vectors have huge potential for clinical in utero gene therapy, considering the disadvantages associated with viral vectors, such as mutagenesis, although additional studies are needed to prove their efficacy for use in clinical settings [8]. Families should be counseled regarding the choice of prenatal therapy or experimental gene therapy [10].

The challenges encountered in the way IUGT becomes a clinical reality include safety, insertional mutagenesis, and oncogenesis. It is also crucial to minimize the risks of fetal intervention, such as fetal demise, injury, and premature birth [9]. Placental gene therapy can bridge the clinical trials of fetal and adult gene therapy. Novel gene editing therapy CRISPR holds a promising place in future IUGT trials [6]. In conclusion, prenatal gene therapy has a wide range of future prospects for it to become a clinical reality [10].

Uses and risks of fetal intrauterine gene therapy

Rationale for Prenatal Gene Therapy

Several aspects of fetal development make treatment delivery more efficient, safe, and long-lasting.

Small fetal size [10]: First, due to the cost and manufacturing constraints associated with gene treatments, the small fetal size allows for effective dosing. A human fetus in the second trimester, the gestational age at which systemic gene therapy is technically viable, weighs around 1% of the weight of a one-year-old child [18].

Accessible proliferating progenitor cells [10]: Second, in the fetus, specific body compartments and cells are more accessible. For example, the fetal blood-brain barrier is permeable, allowing systemic gene therapy to reach a wider range of cell types from the central nervous system. Furthermore, progenitor cells are abundant and generally proliferative in the fetus (essential for successful gene editing through homology-directed repair), increasing the likelihood of propagating a therapeutic correction or gene integration [18].

Tolerogenic fetal immune system [10]: Finally, immune reactions to transgenes and vectors may restrict postnatal gene treatments. Cell and antibody responses to treatments that reduce transgene expression, mediate the loss of genome-edited cells, and exclude individuals from clinical trials have been documented in animal models and humans. However, prenatal delivery studies show that due to the tolerogenic fetal immune system, there is no immunological response to viral transgenes, including Cas9. Substantial research is needed to determine the impact of preexisting maternal antibodies on fetal vector/transgene tolerance [18]. Moreover, treatment should occur before irreversible pathology occurs [10].

Uses

The hazards of prenatal gene therapy are not fully understood, and its efficacy has yet to be demonstrated, as with any other treatment approach. In this regard, according to the NIH’s Recombinant DNA Advisory Committee, prenatal gene therapy should be limited to the following disorders that are linked with substantial morbidity and mortality concerns for the fetus, either in gestation or after birth: (1) Have a bad outcome with existing postnatal therapies or do not have effective postnatal therapy [3]. (2) They are not linked to significant defects that the transplanted gene cannot repair [3]. (3) It has a well-defined genotype/phenotype link and can be identified in pregnancy [3]. (4) Have an in utero gene transfer animal model that mimics human sickness or ailment [3].

Recent gene therapy studies using various vector systems have yielded notable results for hemophilia B, retinal blindness, and SCID, ushering in a new era for a technology that has so far failed to live up to its promise. Candidates for IUGT are likely to have well-characterized monogenic abnormalities that cause severe prenatal or early childhood morbidity. Other disorders include those for which an accurate molecular diagnosis can be achieved before birth and those for which there is no effective postnatal therapy [19].

IUGT might be used to treat a wide range of illnesses. Hemoglobinopathies, for example, have a worldwide carrier frequency of 7%, with alpha-thalassemia major (Bart’s hydrops) being the most lethal without treatment. Other examples include perinatally lethal clotting disorders such as factor VII and X deficiency, lysosomal storage disorders (e.g., mucopolysaccharidoses, Gaucher, Niemann-Pick, and Tay-Sachs diseases) that cause significant perinatal morbidity and mortality, and hereditary blindness such as Leber congenital amaurosis [19]. Preclinical research on prenatal gene transfer is promising. In mouse models of congenital illness, such as hemophilia A and B, congenital blindness, Crigler-Najjar type 1 syndrome, and Pompe disease (glycogen storage disorder type II), fetal treatment of gene therapy has shown phenotypic repair of the condition [3].

Hemophilia

Adult gene therapy techniques have focused on muscle or liver application, with sustained expression of factor IX in adult hemophiliac dogs or mice after intramuscular or intravascular injection of AAV vectors. The AAV serotype is crucial, according to Chao et al. When AAV serotype 1 was injected into mice, it resulted in 10 times larger amounts of canine factor IX than serotype 2. Only short-term and low-level factor IX expression has been reported in clinical studies using AAV2hFIX vectors in patients with hemophilia B, which was likely caused by a cell-mediated immune response to transduced hepatocytes. In adults with hemophilia B, a clinical trial using a self-complementary AAV8 vector serotype is currently underway, which may generate a lower immune response [3].

At 16 days of gestation (term = 22 days), Waddington et al. showed permanent phenotypic correction of immune-competent hemophiliac mice by intravascular injection of a lentivirus vector expressing human factor IX (hFIX) protein [20]. In the treated animals, plasma factor levels remained at 10-15% of normal levels for the rest of their lives. Sabatino et al. demonstrated the induction of tolerance in factor IX-deficient fetal mice following AAV-1-hFIX injection [21].

Hemophilia A and B normally do not show up until after delivery. However, a lack of certain clotting factors can result in life-threatening infant central nervous system hemorrhage. For example, in approximately 20% of patients with a homozygous or compound heterozygous genotype, congenital factor VII deficiency, the most prevalent autosomal bleeding condition, severe, or deadly bleeding occurs. They show up to 1% of normal levels and frequently have central nervous system hemorrhage [3]. A treatment administered during the fetal phase could prevent long-term disease and ensure therapeutic expression for the rest of the patient’s life. Furthermore, even a 1% increase in expression would be considered sufficient to significantly reduce the risk and incidence of spontaneous bleeding [3].

Thalassemia

An adult patient with severe b-thalassemia who had been reliant on monthly transfusions because infancy recently showed the possibility of an ex vivo gene transfer technique for the treatment of thalassemia. He was transfusion-free for at least 21 months after autologous transplantation of gene-modified hematopoietic stem cells using a lentivirus expressing b-globin. The blood hemoglobin level was maintained between 9 and 10 g/dL, with one-third of the blood containing vector-encoded b-globin. The therapeutic impact is attributable to the dominance of a myeloid-based cell clone. The vector’s integration into this clone’s host chromosomal location AT-hook 2 (HMGA2) resulted in transcriptional stimulation of high mobility group protein (HMGA2) production in erythroid cells and truncation of an inhibitory microRNA binding site in this gene. The observed cell growth could be the consequence of *HMGA2* gene expression dysregulation in myeloid stem/progenitor cells, or it could be a random and serendipitous event. More studies are needed to see if this clone is homeostatic or if its growth is a precursor to multistep leukemogenesis, an issue that has been observed in ex vivo autologous stem cell gene therapy for SCID [3].

Cystic Fibrosis

Non-viral gene transfer agents, such as PEI, cationic lipid 67 (GL67), and DNA nanoparticles, are being used in the current clinical study for cystic fibrosis (CF), which can result in a lower immune response. Proof-of-principle investigations in the nasal epithelium reveal a 25% repair of molecular deficiency, and sheep transfected with a human CFTR plasmid complexed with GL67 exhibiting expression of hCFTR [3]. One of the obstacles to efficient gene transfer to the airways in an adult or newborn with CF is that lung damage and inflammation prevent effective gene delivery. This could be overcome if gene therapy is used during the prenatal period when there is no or limited lung damage. Importantly, fetal lungs are fluid-filled, which can make transfection of fluid-filled fetal airways easier. Most cells that grow in the CF airways are basal cells, which would be the ideal target for any gene therapy strategy [3].

Fetal Growth Restriction

Placenta-directed gene therapy induces the production of a therapeutic transgenic protein for a brief period, which might be effective in the treatment of fetal growth restriction (FGR). In preclinical FGR tests, adenoviral vascular endothelial growth factor gene therapy delivered to the placenta via maternal uterine arteries increased fetal growth velocity, and placental insulin-like growth factor gene therapy may enhance glucose and amino acid transport while also increasing fetal development [22]. Gene therapy for obstetric diseases is difficult to translate from the laboratory to the clinic and requires multidisciplinary skills. The risks to the mother and fetus, particularly the possibility of fetal gene transfer, will impact the ethical and societal acceptability of utilizing placenta-directed gene therapy [22].

Neuronal Disorders

The existence of an elevated copy number of the *survival of motor neuron 2* (*SMN2*) gene, a nearly identical copy gene of 4.4.2. spinal muscular atrophy (SMA) *SMN1*, which generates just 10% of the full SMN RNA/protein, can partially protect affected persons. This shows that *SMN2* may have disease-modifying properties and might be a target for disease-modifying gene therapy [3].

Autosomal recessive childhood variants can be divided into three categories based on their severity. Type 0 of the illness affects fetuses in utero, causing decreased fetal movements and arthrogryposis. Type I SMA causes neuronal degeneration and death throughout pregnancy, making prenatal gene therapy an attractive approach. In vitro and in vivo, vectors derived from adenovirus, herpes simplex virus, AAV, and lentivirus can transduce neurons. Neuroprotective factors such as corticotrophin 1 and antiapoptotic proteins such as B-cell lymphoma-extra large (Bcl-xL) can be employed; for example, adenovirus Bcl-xL has been found to prevent neuronal cell death in rat cell cultures. It is also possible to replace the *SMN* gene. Multiple intramuscular injections of a RabG-EIAV lentivirus vector harboring the human *SMN* gene boosted SMN protein levels and decreased motor neuron death in SMA type 1 fibroblasts and SMA mice [3].

SCID

Early in pregnancy, before the immune system matures, prenatal gene therapy could theoretically eliminate the requirement for marrow conditioning or the need for a human leukocyte antigen-matched donor [23]. Prenatal treatment with hematopoietic stem cell transplantation has been attempted for various immunodeficiencies and hemoglobinopathies using intraperitoneal transfer of paternal or maternal hematopoietic cells or fetal livers; however, clinical success was limited to cases of X-linked SCID, where no immune response to transplanted cells could be mounted [23].

Risks of IUGT

The most important risks of IUGT include the ability to disrupt normal fetal development [24]; carry an elevated risk of germline transfer of genetic modification [24]; and induce genotoxicity and/or oncogenesis [24]. These are the primary identified or inferred specific dangers of prenatal gene therapy that generate ethical concerns. Due to the novelty and high degree of uncertainty in the results, the overarching issue is to strike a balance between the ethical norms of beneficence (acting in the patient’s best interests) and nonmaleficence (avoiding injuring the patient) [24].

The ability to disrupt normal fetal development: Due to the change in the accessibility of the vector to tissues and stem cell compartments, as well as the fluctuation in cellular receptor expression throughout early fetal development, Endo et al. group revealed the temporal dependency of transgenic expression after prenatal gene administration in fetal mice. The same group discovered adenomatous abnormalities in the lungs of rat fetuses after prenatal overexpression of fibroblast growth factor 10 [25]. Gonzaga et al. recently discovered that adenovirus-mediated temporary overexpression of transforming growth factor 1 in the embryonic monkey lung during the second or third trimester resulted in severe pulmonary and pleural fibrosis [26].

Carry an elevated risk of germline transfer of the genetic modification: The risk of gene therapy constructing germline transfer is frequently mentioned, with the misunderstanding that it is the goal of fetal gene therapy. It is critical to emphasize that prenatal somatic gene therapy, similar to postnatal gene therapy, is intended solely for the treatment of individual patients, not their children. Any gene therapy study will try to avoid accidentally transferring the therapeutic gene to the treated patient’s germ cells as much as feasible. However, there is a legitimate fear that in utero gene transfer may raise the possibility of unintentional germline gene transfer. Human prenatal gene therapy is unlikely to be performed before seven weeks of pregnancy because germ cells are compartmentalized on the genital ridge during this period [24].

Induce genotoxicity and/or oncogenesis: The incidence of lymphoproliferative illness in five of the 21 children treated with gene therapy using retroviral vectors for the fatal immune deficiency X-SCID shows that the integration of gene therapy vectors can lead to tumor development. This potential for oncogenesis is due to the vector’s random insertion into active genes in the host genome, which can result in oncogene activation or tumor-suppressor gene inactivation. Most of these genes play a role in the regulation of growth and differentiation processes in the body and are particularly active during fetal development. Consequently, they would be ideal integration sites for prenatally administered gene transfer vectors, and in utero, gene therapy might be particularly vulnerable to oncogenesis as a result of their integration with vectors [24].

Ethical issues

In utero gene therapy offers the potential to cure or reduce the severity of a wide range of genetic illnesses for which there are currently no effective therapies. However, there are ethical and practical concerns with prenatal gene therapy that do not arise with postnatal gene therapy. As with any fetal procedure, two patients, the mother and the child, participated in the intervention. The timing of the intervention during the development of the fetus involves critical questions. At the Foreign Forum, a recent panel discussion with international specialists was held at the Society for Fetal Transplantation and Immunology yearly conference, and it summed up many of these [10].

It is important to note that complications such as infection, preterm labor, and fetal loss are theoretically possible with in utero gene therapy; however, strong clinical data are supporting a fetus’s ability to tolerate multiple minimally invasive accesses via intravascular, intraperitoneal, or intra-amniotic routes with very low risks related to the procedure. As previously stated, maternal safety is of paramount importance. Continued animal research is needed to examine the likelihood of maternal exposure to viral vectors and transgenic products as well as any potential maternal immunological response to the injectate. Finally, before the therapeutic use of prenatal in vivo gene augmentation and gene editing, the incidence and effect of viral vector integration and/or off-target consequences must be thoroughly characterized. Fortunately, no substantial germline integration/editing, or off-target effects have been observed in animal investigations thus far. Because these off-target evaluations for in utero gene editing were conducted in a biased manner, future robust and unbiased investigations are needed [10].

## Conclusions

Intrauterine fetal gene therapy provides minimally invasive approaches to preventing genetic disorders. Although various viral and non-viral methods are employed to deliver gene therapies, it is also essential to determine the optimal gestational age and delivery route. Non-viral vectors are advantageous over viral vectors regarding safety and usability. Unfortunately, some vectors, such as naked DNA injection, are restricted in clinical settings due to low transfection efficiency. Nevertheless, research has broadened our knowledge of possible hazards and benefits, like every novel technology we come across. Once prenatal gene therapy has been proven to be safe for treating target disorders, and the strategy of counseling families and implementing in utero gene therapy has been perfected, it can be considered for broader use. We believe that fetal intrauterine gene therapy is the future of fetal and neonatal medicine to improve quality of life and potentially cure monogenetic disorders before irreversible pathology occurs. Soon, humankind will start accepting progress and be a part of it.
